# Cervical Spinal Meningeal Melanocytoma Presenting as Intracranial Superficial Siderosis

**DOI:** 10.1155/2015/674868

**Published:** 2015-12-07

**Authors:** Savitha Srirama Jayamma, Seema Sud, TBS Buxi, VS Madan, Ashish Goyal, Shashi Dhawan

**Affiliations:** ^1^Department of CT and MRI, Sir Ganga Ram Hospital, New Delhi 110060, India; ^2^Department of Neurosurgery, Sir Ganga Ram Hospital, New Delhi 110060, India; ^3^Department of Pathology, Sir Ganga Ram Hospital, New Delhi 110060, India

## Abstract

Meningeal melanocytoma is a rare pigmented tumor of the leptomeningeal melanocytes. This rare entity results in diagnostic difficulty in imaging unless clinical and histopathology correlation is performed. In this case report, we describe a case of meningeal melanocytoma of the cervical region presenting with superficial siderosis. Extensive neuroradiological examination is necessary to locate the source of the bleeding in such patients. Usually, the patient will be cured by the complete surgical excision of the lesion.

## 1. Introduction

Meningeal melanocytoma is a rare tumour which arises from leptomeningeal melanocytes. They are considered to be the benign end of the neoplastic lesions of the leptomeningeal melanocytes. The preoperative diagnosis of the meningeal melanocytoma is often a diagnostic challenge as the clinical and neurological features are often nonspecific. These tumors have better prognosis than their malignant counterparts [1]. They can present as diffuse disseminations within the subarachnoid spaces, space occupying solid masses within the central nervous system. They present as slowly growing mass lesions with focal neurological deficits due to the mass effect on the adjacent tissues [2]. Here, the authors describe a case of meningeal melanocytoma of the cervical spine presenting as superficial siderosis of the central nervous system that was treated successfully with complete excision of the lesion by cervical laminectomy from C3 to C5 level. Local recurrence and leptomeningeal spread of these tumors secondary to the malignant transformation are well reported in the literature [3].

## 2. Case Report

**Figure 1:**
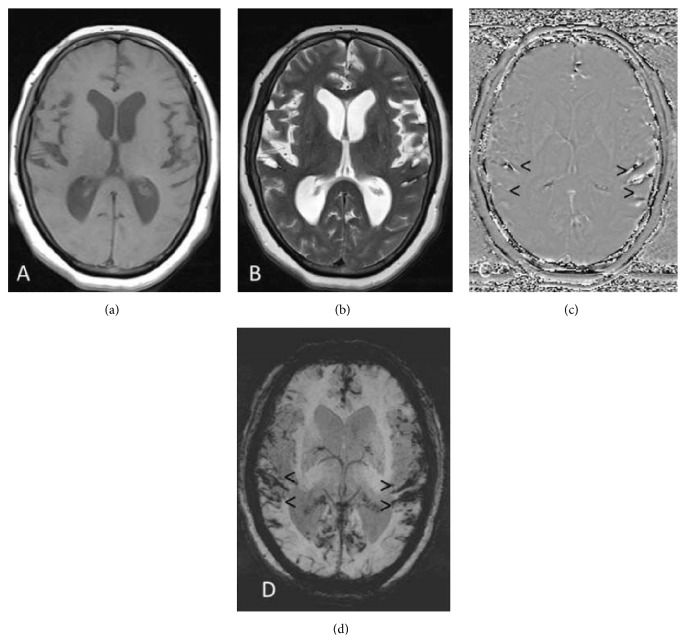
Axial sections of the brain: (a) T1 weighted and (b) T2 weighted images showing mild effacement of the sulci. Positive phase shift and blooming along the sulcal spaces (arrow heads) on (c) phase and (d) MIP (susceptibility weighted) images suggestive of superficial siderosis.

A 62-year-old hypertensive, obese, diabetic, male patient with short neck presented with episodic falls, difficulty in walking, and brief loss of consciousness to our hospital. Weakness of both hands and progressive weakness of all four limbs were present since 1 week. The patient was diagnosed as complex partial seizures clinically. There was no history of trauma at the presentation or in the past. The patient had a history of subarachnoid haemorrhage 6 months back for which conservative management was done elsewhere. Magnetic resonance (MR) imaging of the brain showed mild effacement of the sulcal spaces on T1 weighted images (T1WI) and T2 weighted images (T2WI) (Figures 1(a) and 1(b)). There was a positive phase shift and blooming along the sulcal spaces on phase contrast and maximum intensity projection (MIP) susceptibility weighted images (arrow heads in Figures 1(c) and 1(d)) suggestive of superficial siderosis. There was no evidence to suggest the possible source of haemorrhage in the MR images of the brain. The remainder of general physical and systemic clinical examination was also unremarkable. Screening of the spine was done to locate the possible source of the bleeding leading to superficial siderosis.

**Figure 2:**
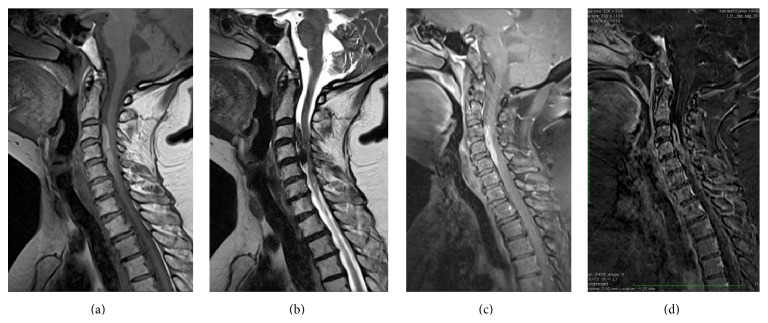
Sagittal sections of the cervical vertebral column: (a) T2 weighted and (b) T1 weighted images showing an intradural extramedullary mass which appears hypointense on T2 weighted image and hyperintense on T1 weighted image. (c) Postcontrast fat saturated T1 weighted and (d) subtracted contrast image showing mild peripheral heterogeneous enhancement of the tumor since the true enhancement pattern was obscured by the strong T1 hyperintensity of the mass on unenhanced images.

**Figure 3:**
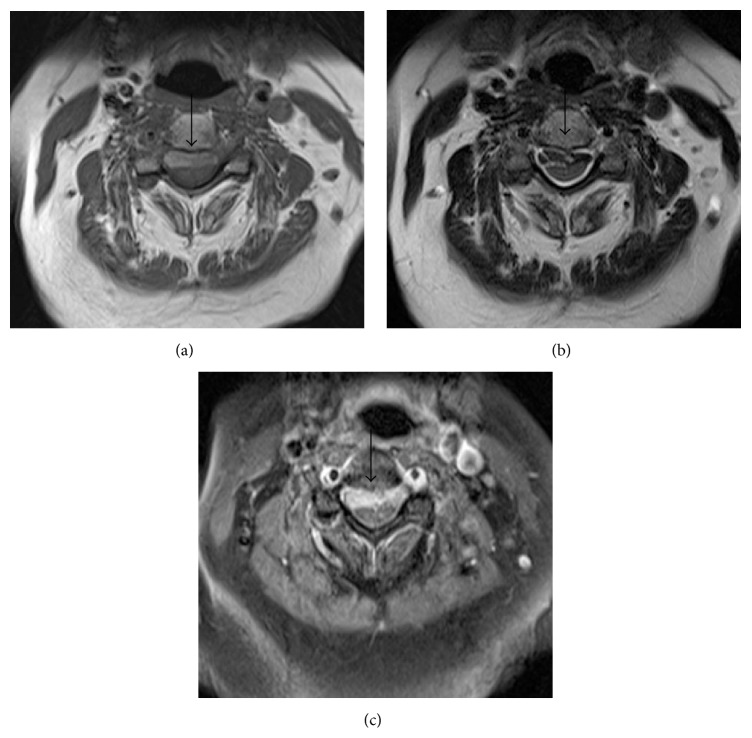
Axial images of the cervical vertebral column: (a) T1 weighted and (b) T2 weighted images showing an intradural extramedullary mass (arrow) occupying the anterior intradural space, compressing and displacing the spinal cord posteriorly. (c) On postcontrast fat saturated T1 weighted images the lesion shows peripheral heterogeneous enhancement.

**Figure 4:**
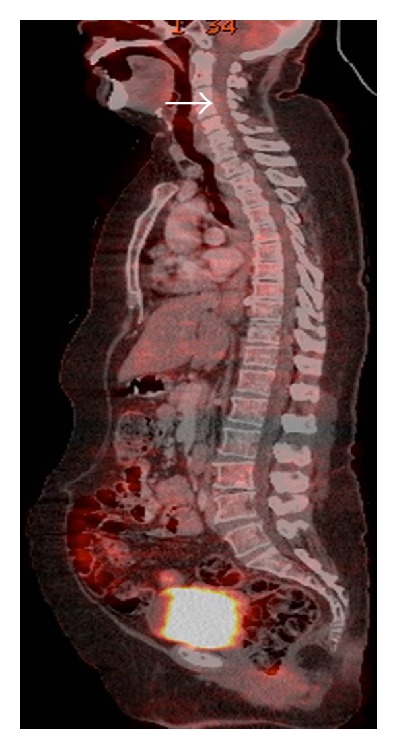
PET image showing moderately FDG avid intradural mass lesion (arrow).

**Figure 5:**
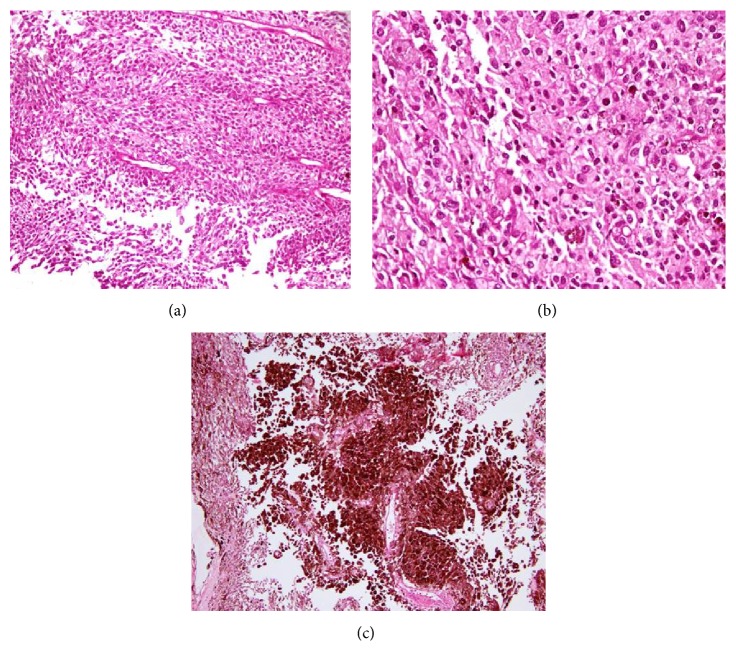
Histopathological examination. (a) Diffusely pigmented tumor with peritheliomatous arrangement which obscured the cytological details (H and E 40x). (b) After bleaching, tumor had pleomorphic round to oval nuclei with inconspicuous nucleoli and moderate amount of cytoplasm (H and E 40x). (c) Tumor on higher magnification had mild nuclear pleomorphism, moderate to abundant cytoplasm with few containing blackish brown pigment. No significant increase in mitosis was seen (H and E 200x).

Cervical spinal magnetic resonance imaging (MRI) revealed an intradural extramedullary mass occupying the anterior intradural space which displayed hypointense signal on T2WI and hyperintense signal on T1WI (Figures 2(a), 2(b), 3(a), and 3(b)). Contrast enhancement of the mass was not evidently revealed by visual assessment on postcontrast fat saturated T1 weighted images (Figure 2(c)) due to strong T1 hyperintensity of the mass on unenhanced images. Mild peripheral heterogeneous enhancement of the tumor was verified by the subtracted images (Figure 2(d)). The lesion was compressing and displacing the spinal cord posteriorly (arrow in Figures 3(a), 3(b), and 3(c)). The PET CT images showed moderately FDG avid extramedullary intradural mass (arrow in Figure 4) and no other foci of FDG avidity were noted elsewhere in the body. The patient underwent C3–C5 laminectomy and excision of the intradural extramedullary mass lesion. The Lesion was placed anterior to the cord and was firm, smooth surfaced, and blackish in colour. Retrospectively, examinations of the skin and the fundus of the eye did not reveal any melanotic lesions. Hence the lesion was treated as primary cervical spinal melanocytoma which was confirmed on histopathological examination which revealed diffusely pigmented tumor with peritheliomatous arrangement which obscured the cytological details (H and E 40x) (Figure 5(a)). After bleaching, tumor had pleomorphic round to oval nuclei with inconspicuous nucleoli and moderate amount of cytoplasm (H and E 40x) (Figure 5(b)). Tumor on higher magnification had a mild nuclear pleomorphism, moderate to abundant cytoplasm with few containing blackish brown pigment. No significant increase in mitosis (<1 mitosis per 10 high power fields) was seen (H and E 200x) (Figure 5(c)). The proliferation index Ki-67 was low (<1%) which is diagnostic of melanocytomas. The patient improved symptomatically in the postoperative period.

## 3. Discussion

Melanocytes are cells of neural crest origin and are normally found in the leptomeningeal layers. Melanocytes may cause primary melanocytic neoplasms which can be classified as melanocytoma (benign end of the spectrum), intermediate grade melanocytic neoplasms, and primary malignant melanoma. These neoplasms can be differentiated histologically based on the identification of mitotic activity, cytological atypia, necrosis, and invasion of the adjacent structures [2]. Melanocytoma can occur along the neural axis most commonly in posterior fossa, adjacent to the cranial nerve nuclei, and Meckel's cave and in the foramen magnum. Within the spine, it present as intradural extramedullary masses, mostly found in the upper cervical region, as the melanocytes are most concentrated in this region [4]. They tend to present in fourth and fifth decades of life. Spinal melanocytoma present with weakness, sensory deficits, and progressive pain. Subarachnoid haemorrhage can be a rare presentation of these tumors [1]. Meningeal melanocytoma was introduced as a primary melanocytic lesion from the leptomeninges with benign clinical and pathologic features by Limas and Tio in 1972 [5]. There are few case reports of meningeal melanocytoma from the spinal column [6, 7] and cerebellopontine region [8] presenting as superficial siderosis of the central nervous system in the literature.

On computed tomography, these lesions present as well-defined, isodense to hyperdense, homogenous, and contrast enhancing mass lesions. The MRI appearance of meningeal melanocytoma is variable, depending on the amount of melanin content present. Signal characteristics include isointense or hyperintense on T1 weighted images and isointense or hypointense on T2 weighted images which give a heterogeneous enhancement on postcontrast images and show blooming of low signal on gradient images [9]. The contrast enhanced T1 weighted images and subtracted contrast enhanced images should be obtained as these tumors show T1W hyperintensity.

Complete excision is the treatment of choice; however this is often not possible as intraoperative haemorrhage may be severe. Furthermore, local recurrence has been reported even after gross total removal. Risk of tumour recurrence is noted even after complete excision; hence, adjuvant radiation therapy is advised in cases of both complete and incomplete resection [10].

Melanocytoma should be considered in the differential diagnosis of the pigmented tumors which include meningeal meningioma, melanotic schwannoma, and metastatic and primary malignant melanoma [11, 12]. Radiologically, the differential diagnosis of these primary pigmented lesions is difficult due to their similar appearances on CT and MR imaging, thus necessitating further diagnostic confirmation. Tumor calcification and hyperostosis of adjacent bones are visualised in melanocytomas; however, a lack of these signs obviously does not exclude the presence of a meningioma. Small schwannomas usually show uniform contrast enhancement, whereas larger schwannomas may be heterogeneous [2]. Histopathological examination is the most crucial method of differentiating primary melanocytoma from other pigmented lesions of the leptomeninges.

Due to abundant melanin production, meningeal melanocytomas appear dark brown to black on gross examination. They feature pigmented spindle cells growing in tight nests. Cytologically, tumor cells are bland with less pleomorphism and indistinct nucleoli. These must be distinguished from intermediate grade melanocytic neoplasms and malignant melanoma owing to the same cell of origin. Intermediate grade melanocytic neoplasms should be considered when bland cytology with some CNS invasion, mitotic activity (0–3 mitoses per 10 high power fields), and Ki-67 labelling index ranging from 1–4 (% of nuclei) is seen. Features in favour of malignant melanoma include high mitotic activity (2–15 mitoses per 10 high power fields), central nervous system invasion, tumor necrosis, Ki-67 labelling index ranging from 2–15 (% of nuclei), and cellular and nuclear atypia [2]. Melanocytomas are typically immunoreactive for S-100 protein and HMB-45. A positive immunostain for epithelial membrane antibody can confirm the diagnosis of melanotic meningioma. Melanotic schwannomas show considerable histologic overlap with melanocytomas and both tumors are immunoreactive for S-100 protein, therefore making the distinction between these tumors difficult. However, immunohistochemical staining for HMB-45 to detect the melanocytic origin can help to reach an accurate diagnosis as in our case [2]. The histologic differentiation between malignant melanoma and melanocytoma is difficult. The lack of mitotic activity, presence of large nucleoli, the lack of nuclear pleomorphism, hyperchromicity, and slow growth are the pointers toward the diagnosis of melanocytoma [1]. Although extremely rare, meningeal melanocytoma may be associated with histological benign leptomeningeal spread and aggressive clinical course in spite of absence of malignant transformation.

In summary, although the imaging might not differentiate the pigmented lesions very well, it helps in early identification of the complications like diffuse benign spread and recurrence. Making a correct differential diagnosis and being alert to the unusual presentation of meningeal melanocytoma as superficial siderosis will result in appropriate therapy as well as identification of the complications in the follow-up.

## References

[1] Painter T. J., Chaljub G., Sethi R., Singh H., Gelman B. (2000). Intracranial and intraspinal meningeal melanocytosis. *American Journal of Neuroradiology*.

[2] Brat D. J., Perry A., Brat D. J. (2010). Melanocytic neoplasms of the central nervous system. *Practical Surgical Neuropathology: A Diagnostic Approach*.

[3] Roser F., Nakamura M., Brandis A., Hans V., Vorkapic P., Samii M. (2004). Transition from meningeal melanocytoma to primary cerebral melanoma. Case report. *Journal of Neurosurgery*.

[4] Chacko G., Rajshekhar V. (2008). Thoracic intramedullary melanocytoma with long-term follow-up: case report. *Journal of Neurosurgery: Spine*.

[5] Limas C., Tio F. O. (1972). Meningeal melanocytoma (‘melanotic meningioma’). Its melanocytic origin as revealed by electron microscopy. *Cancer*.

[6] Matsumoto S., Kang Y., Sato S. (1998). Spinal meningeal melanocytoma presenting with superficial siderosis of the central nervous system. Case report and review of the literature. *Journal of Neurosurgery*.

[7] Das A., Ratnagopal P., Puvanendran K., Teo J. G. C. (2001). Spinal meningeal melanocytoma with hydrocephalus and intracranial superficial siderosis. *Internal Medicine Journal*.

[8] Vreto G., Rroji A., Xhumari A., Leka L., Rakacolli M., Petrela M. (2011). Meningeal melanocytoma of the cerebellopontine angle as the unusual cause of superficial siderosis. *Neuroradiology*.

[9] Chen C. J., Hsu Y. I., Ho Y. S., Hsu Y. H., Wang L. J., Wong Y. C. (1997). Intracranial meningeal melanocytoma. CT and MRI. *Neuroradiology*.

[10] Kim O. H., Kim S. J., Choo H. J. (2013). Spinal meningeal melanocytoma with benign histology showing leptomeningeal spread: case report. *Korean Journal of Radiology*.

[11] Litofsky N. S., Zee C.-S., Breeze R. E., Chandrasoma P. T., Leavens M. E., Winston K. R. (1992). Meningeal melanocytoma: diagnostic criteria for a rare lesion. *Neurosurgery*.

[12] Tatagiba M., Boker D.-K., Brandis A. (1992). Meningeal melanocytoma of the C8 nerve root: case report. *Neurosurgery*.

